# Atorvastatin and diacerein reduce insulin resistance and increase disease tolerance in rats with sepsis

**DOI:** 10.1186/s12950-018-0184-9

**Published:** 2018-05-09

**Authors:** K. L. C. da Silva, A. P. Camacho, F. C. Mittestainer, B. M. Carvalho, A. Santos, D. Guadagnini, A. G. Oliveira, M. J. A. Saad

**Affiliations:** 1 Department of Internal Medicine, State University of Campinas, Campinas, SP Brazil; 2 Department of Biology Science, Federal University of Pernambuco, Recife, PE Brazil; 3 Department of Physical Education, São Paulo State University (UNESP), Bioscience Institute, Rio Claro, SP Brazil; 4 Departamento de Clínica Médica, FCM-UNICAMP, Cidade Universitária Zeferino Vaz, Campinas, SP 13083-887 Brazil

**Keywords:** Statin, Diacerein, Insulin resistance, Sepsis

## Abstract

**Background:**

Sepsis is one of the leading causes of death among hospitalized patients. At the onset of this condition, there is an over-production of pro-inflammatory mediators that contribute to organ failure and death. The excess production of pro-inflammatory mediators also impairs insulin signaling, which may be a pathophysiological tissue marker of proinflammatory cytokine action before organ failure. Statins and diacerein have pleiotropic effects, such as the blockage of inflammatory signaling pathways, suggesting that these drugs may be an attractive therapeutic or prophylactic strategy against sepsis. The aim of the present study was to investigate whether a statin or diacerein can improve insulin signaling, disease tolerance and survival in sepsis by inhibiting inflammatory pathways.

**Methods:**

We investigated the effect of these drugs on survival, tissue insulin signaling and inflammatory pathways in the liver and muscle of rats with sepsis induced by cecal ligation and puncture (CLP).

**Results:**

The results showed that administration of medications, with anti-inflammatory ability, to septic animals increased survival and improved disease tolerance and insulin resistance in the liver and muscle. The treatment also attenuated ER stress, NF-κB, JNK activation and restored glucose-6-phosphatase (G6Pase) levels in the liver.

**Conclusions:**

Our results indicate that atorvastatin and diacerein treatment can modulate inflammatory pathways and, in parallel, attenuate insulin resistance in sepsis. Since these two drugs have safety profiles and minimal side effects, we suggest that these drugs may be alternative therapies for the prevention or therapies for the treatment of insulin resistance in sepsis, which could potentially reduce mortality in patients with sepsis.

**Electronic supplementary material:**

The online version of this article (10.1186/s12950-018-0184-9) contains supplementary material, which is available to authorized users.

## Background

Sepsis, defined as a life-threatening organ dysfunction caused by a dysregulated host response to infection [1], is a prevalent disease and figures as one of the leading causes of death among hospitalized patients [2]. During the onset of sepsis, there is an over-production of pro-inflammatory mediators [3], which contribute to organ failure, and death. The excess production of pro-inflammatory mediators results in a significant pro-inflammatory state, which in turn reduces insulin sensitivity [4–10]. This insulin resistance may be a pathophysiological tissue marker of proinflammatory cytokine action before organ failure.

During sepsis, lipopolysaccharide-LPS (a bacterial component) and endogenous cytokines may induce inflammation and insulin resistance, through endoplasmic reticulum (ER) stress and activation of the NF-κB and JNK pathways [8, 11, 12]. The molecular mechanism by which insulin resistance is induced is through serine phosphorylation of the insulin receptor substrate 1 (IRS-1), thus impairing insulin action. This insulin resistance, in the liver and muscle, characterized by a down-regulation of insulin signaling may unveil the overreaction at the tissue level, and may also be used to predict the effectiveness of treatment.

The pathologic outcome of sepsis is related to pathogen aggressiveness but is also a direct consequence of the metabolic dysfunction and damage occurring in the tissues. A defense strategy that does not directly interfere with the pathogen load, but limits the pathologic outcome of the infection is called disease tolerance. This process works to maintain the homeostatic parameters compatible with host survival.

Over the last few decades, the knowledge about sepsis, inflammation and organ failure has been increasing [13, 14], but pharmacological interventions to induce disease tolerance are still not effective in reducing the high mortality rate. Recently, a disappointing result from a phase 3 clinical trial with an MD2-TLR4 antagonist [15] showed that this antagonist did not provide beneficial effects in sepsis, when compared to placebo. It is not easy to reconcile a clinical study like this with a preclinical result in rats, but it is important to mention that the time of treatment and the specific or nonspecific blockage of a single inflammatory pathway are critical in the interpretation of the results. In this regard, we believe that there is a therapeutic window, and the use of more broad anti-inflammatory drugs might have beneficial effects in severe sepsis.

Recently, knowledge about statins, a class of hypolipidemic drugs with pleiotropic effects, such as anti-inflammatory properties, suggest that these drugs are an attractive therapeutic or prophylactic strategy for the treatment of sepsis [2, 3, 16]. Epidemiological evidence suggests that statins may reduce mortality rates in sepsis [17–24]. However, the molecular mechanisms by which statins induce this protective effect have not yet been investigated. Another drug that has anti-inflammatory effects is Diacerein (4,5-diacetoxy-9,10-dihydro-9,10-dioco-2-anthracenecarboxylic acid), which is an anthraquinone [13] that has been used in the treatment of osteoarthritis [25]. The active metabolite of diacerein – Rhein – blocks the synthesis of TNF-α, IL-6, and IL-1β [26–30]. There is also evidence that diacerein inhibits the activation of the NF-κB pathway [31], suggesting that this drug could improve insulin resistance in sepsis.

This study aimed to investigate whether a statin or diacerein can improve insulin signaling, contribute to disease tolerance and improve survival in sepsis, by reducing inflammatory tissue pathways.

## Methods

### Materials

The antibodies used were anti-Akt (rabbit polyclonal, #9272), anti-phospho-Akt (rabbit polyclonal, #9271), anti-β-Actin (rabbit polyclonal, #4967), anti-IRS1 (rabbit polyclonal, #2382) and anti-phospho-IRS1^ser307^ (rabbit polyclonal, #2381) from Cell Signaling Technology (Beverly, MA, USA); anti-JNK (rabbit polyclonal, SC1648), anti-phopho-JNK (mouse monoclonal, SC6254), anti-G6pase (goat polyclonal, SC 27198) and anti-α-Tubulin (mouse monoclonal, SC8035) from Santa Cruz Technology (Santa Cruz, CA, USA). Atorvastatin was obtained from Pfizer (Loughbeg, County Cork, Ireland) and Diacerein was kindly provided by TRB-Pharma (Campinas, Brazil). Human recombinant insulin was from Eli Lilly and Co. (Indianapolis, Indiana, USA). Routine reagents were purchased from Sigma Chemical Co. (St. Louis, MO, USA) unless specified elsewhere.

### Animals

Male *Wistar* rats (8 weeks old; obtained from the State University of Campinas Central Breeding Center) were stored under a 12/12-h light/dark cycle in the controlled room (humidity ~ 55 ± % and temperature ~ 22 ± 3 °C) with assess to food and water ad libitum. The animals were randomly separated into the following groups: sham-operated animals (Control group), placebo-treated (saline) sepsis (Sepsis group), atorvastatin-treated sepsis (Atorvastatin group), and diacerein-treated sepsis (Diacerein group). All animal protocols of this study were approved by the Animal Care and Use Committee at State University of Campinas (CEUA/UNICAMP 1267-1), and they are by international guidelines for the care and use of experimental animals.

### Surgical procedures

Cecal ligation and puncture (CLP) were executed, as described before [32], to induce a sepsis model. The rats were anesthetized by using ketamine (80 mg/kg) and xylazine (15 mg/kg). The cecum was attached right below the ileocecal valve carefully to prevent obstruction of either ileum or colon. The cecum was exposed to a four “through-and-through” punctures (20-gauge needle). The abdominal incision was closed in layers. The sham-operated rat underwent the same procedure, except for ligation and perforation of the cecum. All processes were carried out under sterile conditions. Three hours after the induction of sepsis and every 24 h, rats received atorvastatin (10 mg/kg/day) or diacerein (100 mg/kg/day) or an equivalent volume of the vehicle by oral gavage. The dose of atorvastatin was chosen based on a previous study [33] and the higher metabolic rate of the drug in rodents [34]. The diacerein dose was selected based on a previous study [35].

### Sepsis-survival studies

*Wistar* rats (*n* = 20 per group) were induced to sepsis, as previously described and allowed to recover. Then, these animals were treated with diacerein, atorvastatin or placebo 3 h after surgery and then once per day, and were observed every 4 h for 1-week, than every 12 h until death or for 15 days.

### Insulin tolerance test (ITT)

Insulin sensitivity was investigated through the insulin tolerance test (ITT) at 24 h after surgery, as described [36]. Insulin (1.5 U/kg) was administered by i.p. injection and blood samples were collected at 0, 5, 10, 15, 20, 25, and 30 min to determine serum glucose. The constant rate of glucose disappearance (Kitt) was calculated as previously described [37].

### ELISA assays

Samples of blood were collected 24 h after surgery. To prepare the serum, whole blood was directly drawn into a centrifuge tube without anti-coagulant. The blood clot was formed after 30 min at room temperature, and then centrifuged, transferred and stored in separate tubes at − 80 °C for further ELISA analysis. IL-6, and TNF-α were determined using commercially available ELISA kits (Pierce Biotechnology Inc., Rockford, IL, USA) in serum. Part of the tissues (liver and muscle) obtained as further described in section “*Tissue Extraction and Immunoblotting*” was immediately frozen in liquid nitrogen and stored at − 80 °C until NF-κB nuclear extraction and analysis using commercially available ELISA kit (Thermo Fisher Scientific Pierce Biotechnology Inc., Rockford, IL) following the manufacturer’s instructions.

### Tissue extraction and immunoblotting

After 24 h from surgery, rats were anesthetized by using of sodium thiopental (40 mg/Kg) and used as soon as anesthesia was guaranteed by the loss of pedal and corneal reflexes. Saline (0.1 ml) or insulin were injected (3.8 U/Kg IP) and the liver fragment removed after 30 s, and muscle was obtained after 90 s of injection, and then all tissues were minced coarsely and homogenized immediately in extraction buffer, centrifuged (15.10^3^ rcf for 30 min at 4 °C) as done previously [38], and stored at -80 °C until further use. The tissue extracts were subjected to SDS-PAGE and immunoblotting, as previously described [36].

### qPCR

Part of the tissues (liver and muscle) obtained as described in section “*Tissue Extraction and Immunoblotting*” was immediately frozen in liquid nitrogen and stored at -80 °C until total RNA extraction. Total RNA was extracted using the RNeasy Mini Kit (Germantown, MD, USA) according to the manufacturer’s instructions. The qPCR was performed in QuantStudio 6 Flex Real-Time PCR System (Thermo Fisher Scientific) using SybrGreen PCR Master Mix (Thermo Fisher Scientific, Carlsbad, CA, USA), (Primers described in Additional file 1: Table S1) and analyzed using the ΔΔCt method. Relative gene expression was normalized to β-actin.

### Statistical analysis

Data are exhibited as a mean ± standard error of the mean (SEM). The results of blots are presented as direct comparisons of bands and quantified by optical densitometry (UN SCAN IT, Silk Scientific Inc., Orem, UT, USA). Multiple comparisons were tested by one-way ANOVA, followed by Tukey’s post hoc test, with the significance level set at *P* < 0.05 using GraphPad Prism software (La Jolla, CA, USA). The overall difference in survival rate was determined by the Kaplan-Meier test followed by a log-rank test.

## Results

### Atorvastatin and Diacerein improve survival in septic rats

**Fig. 1:**
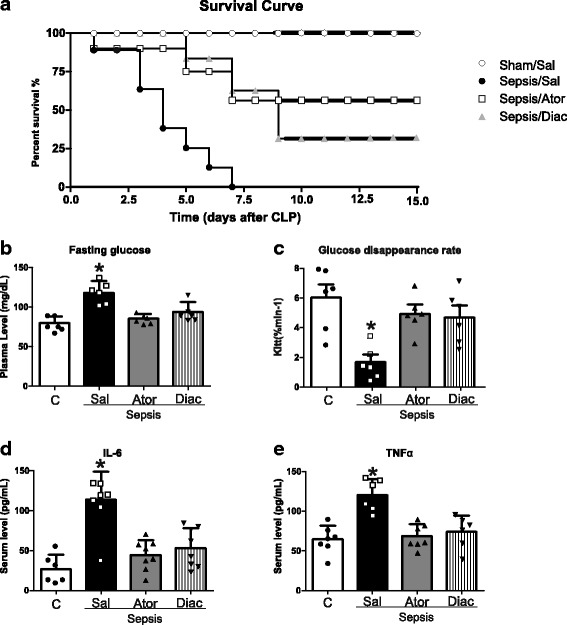
Effect Atorvastatin and Diacerein survival in CLP sepsis model. Male *Wistars* rats, 8 weeks old, received saline (Sham/Sal, *n*  =  20), (Sepsis/Sal, *n*  =  20) or atorvastatin 10 mg/kg (Sepsis/Ator, *n*  =  20), or diacerein 100 mg/kg (Sepsis/Diac *n*  =  20) 3 h after surgery and once a day following CLP. Survival of the rats was monitored at intervals of 4 h for 1-week, than every 12 h until day 15. The overall difference in survival rate between the groups with and without atorvastatin was significant (*P* < 0.0001). The overall difference in survival rate between the groups with and without diacerein was significant (*P* < 0.0001) (**a**). Fasting blood glucose (**b**). Glucose disappearance rate (**c**). Serum levels of IL-6 (**d**) and TNF-α (**e**). Data are presented as means ± SEM of six rats per group (except for survival curve). *P < 0.05 (Sepsis saline vs. all others groups)

To test the hypothesis that these two drugs with anti-inflammatory activity can decrease sepsis mortality, we monitored the survival in septic and sham-operated animals. The results showed a significant improvement in the survival curves after atorvastatin (*P* < 0.0001) or diacerein (*P* < 0.0001) treatment in septic animals (Fig. 1a), when compared to septic animals that received a placebo. However, no significant difference was observed between the two treatments in the sepsis-induced or sham-operated animals.

### Effect of atorvastatin and diacerein on plasma glucose levels, insulin tolerance test and serum levels of IL-6, and TNF-α

Plasma glucose levels were higher in septic animals, and both atorvastatin and diacerein normalized this parameter (Fig. 1b). Along the same line, septic animals displayed a reduced Kitt, which is indicative of insulin resistance, and both drugs improved insulin sensitivity (Fig. 1c). With regards to cytokine levels, septic animals displayed higher serum levels of IL-6 and TNF-α than the sham-operated rats. After atorvastatin or diacerein treatment, a significant decrease was observed in the circulating levels of all of the cytokines evaluated (Fig. 1d-e). Taken together these results demonstrated that both treatments were able to improve the metabolic and inflammatory profile of septic animals.

### Atorvastatin or diacerein improves insulin signaling in septic animals

**Fig. 2:**
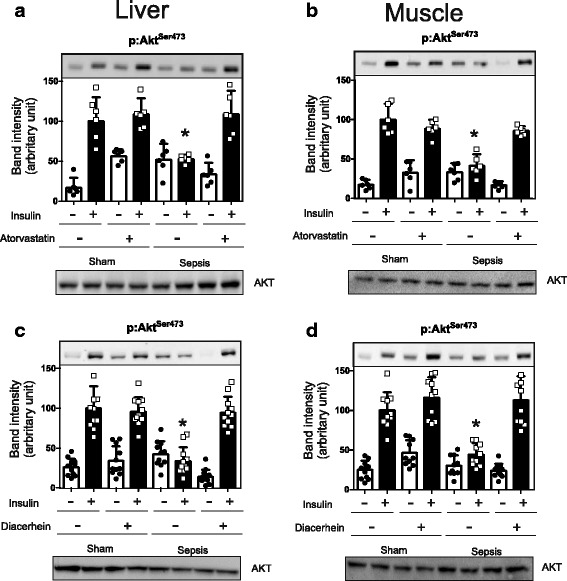
Effects of Atorvastatin and Diacerein treatment on insulin signaling in the CLP rat. Insulin-induced protein expression of Akt serine phosphorylation with Atorvatatin or Diacerein treatment (**a**-**d** upper panels). **a**- Atorvastatin/Liver; **b**- Atorvastatin/Muscle; **c**- Diacerein /Liver and **d**- Diacerein/Muscle. In this case, blots quantification were normalized with total AKT protein expression (**a**-**d**, lower panels). The amount of protein loaded was 30 μg. Data are presented as means +/− SEM from 6 rats per group. *P < 0.05 (Sepsis/Sal vs. all others groups). IB, immunoblotting; p, phosphorylated

In the sepsis group, insulin-induced Akt serine phosphorylation in the liver and muscle was blunted when compared with the sham-operated rats, and both atorvastatin and diacerein were able to significantly increase Akt phosphorylation in these tissues (Fig. 2a-d). However, there was no significant change in the protein expression levels of Akt observed in the liver or muscle, among all of the groups studied (Fig. 2a-d).

### Atorvastatin and diacerein attenuate sepsis-induced inflammatory changes

**Fig. 3:**
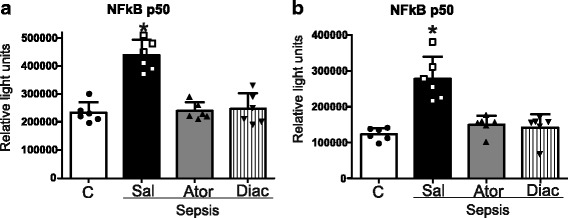
NFkB activation in nuclear fractions. NFκB activation in nuclear fractions of liver (**a**) and muscle (**b**) of sham and septic rats were given saline or atorvastatin or diacerein . Data are presented as means ± SEM from 6 rats per group. *P < 0.05 (Sepsis/Sal vs. all others groups). C: Sham/Saline; Sal: Saline: Ator: Atorvastatin; Diac: Diacerein

**Fig. 4:**
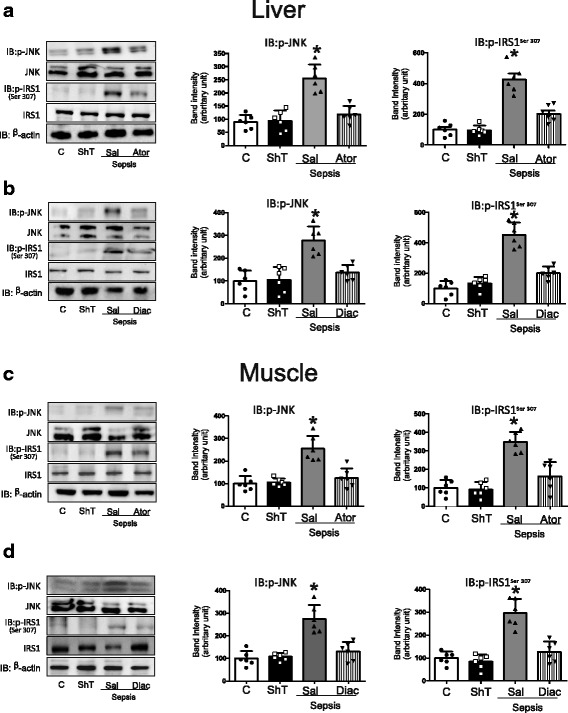
Atorvastatin or diacerein attenuate JNK and IRS phosphorylation in liver and muscle of septic animals. Phosphorylation of JNK in liver (**a**-**b**), muscle (**c**-**d**) after Atorvatatin or Diacerein treatment of sham and septic rats. Total protein expression of JNK. Serine 307 Phosphorylation of IRS1 in liver (**a**-**b**), muscle (**c**-**d**) after Atorvatatin or Diacerein treatment of septic rats. Total protein expression of IRS-1. The amount of protein loaded was 30 μg. Data are presented as means ± SEM from 6 rats per group. *P < 0.05 (Sepsis/Sal vs. all others groups). IB, immunoblot; C: Sham/Saline; Sal: Saline: Ator: Atorvastatin; Diac: Diacerein; ShT: Sham treatment

It is well known that the proinflammatory pathways are activated in sepsis, including the IKKβ/NF-κB and the JNK pathways. Thus, we examined the anti-inflammatory effects of atorvastatin and diacerein on the NF-κB pathway using an activity assay. The results showed that NF-κB was activated in sepsis, and both drugs were able to attenuate this activation (Fig. 3a-b). Next, we evaluated JNK phosphorylation levels, which is an indicator of its activity. The results showed that in the liver and muscle of septic rats there was an increase in JNK phosphorylation, and the treatment with atorvastatin or diacerein blocked this activation in both tissues (Fig. 4a-d). Since this serine kinase can induce IRS-1 serine phosphorylation, we also investigated the effects of atorvastatin or diacerein on IRS-1^ser307^ phosphorylation levels in the liver and muscle of septic animals. The results showed that there was an increase in IRS-1 serine phosphorylation levels in the liver and muscle of septic rats, and treatment with both drugs reduced the serine phosphorylation of IRS-1 (Fig. 4a-d).

### Atorvastatin and diacerein treatment lowers endoplasmatic reticulum stress in septic rats

**Fig. 5:**
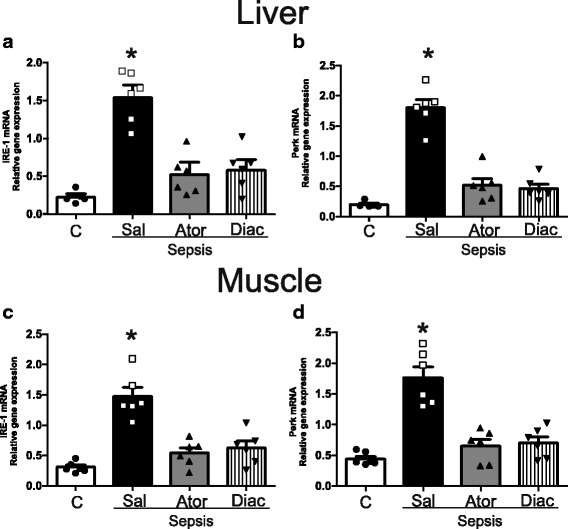
Effects of Atorvastatin or Diacerein administration on the expression of gene PERK and IRE1 in septic rats. Determination of IRE1 and PERK mRNA expression by qPCR in the liver (**a**-**b**) and muscles (**c**-**d**) after Atorvatatin or Diacerein treatment of septic rats. Data are presented as means ± SEM from 6 rats per group. *P < 0.05 (Sepsis/Sal vs. all others groups). C: Sham/Saline; Sal: Saline: Ator: Atorvastatin; Diac: Diacerein

It is well established that sepsis can induce ER stress and the unfolded protein response (UPR). In this regard, we investigated the effect of sepsis on genes that encode for proteins that reflect the UPR response. Our data showed that in sepsis there were increased mRNA levels of PKR-like endoplasmic reticulum kinase (PERK) and inositol-requiring enzyme 1 (IRE1), and the treatment with atorvastatin or diacerein significantly reduced the expression of these genes (Fig. 5a-d).

### Atorvastatin and diacerein restores glucose-6-phosphatase (G6Pase) in the liver of septic animals

**Fig. 6:**
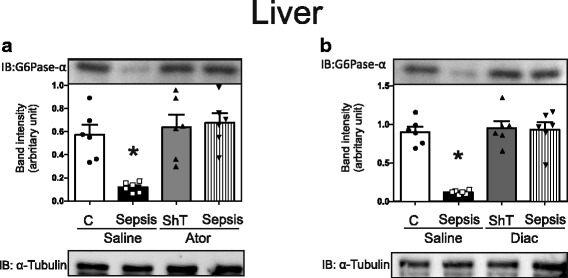
Atorvastatin (**a**) and diacerein (**b**) restore G6Pase in liver of septic animals. Protein expression of G6Pase in the liver after Atorvatatin or Diacerein treatment of sham and septic rats. The amount of protein loaded was 60 μg. Data are presented as means ± SEM from 6 rats per group. *P < 0.05 (Sepsis/Sal vs. all others groups). IB, immunoblot; C: Sham/Saline; Ator: Atorvastatin; Diac: Diacerein, ShT: Sham treatment

The insulin resistance in the liver and peripheral tissues in sepsis is an adaptive response accompanied, at least in the initial phase, by mild hyperglycemia as observed in the present study. However, during the evolution of sepsis, the development of hypoglycemia should be prevented to improve disease tolerance [39]. It is well known that in septic animals there is an increase in TLR4 and TLR2 signaling and that ER stress could contribute to reduced insulin signaling in the liver. Our data showed that the activation of the NF-κB and JNK pathways, which are downstream from these TRLs, in the liver of septic animals, is also accompanied by a reduction in the protein expression of G6Pase. This reduction in G6Pase protein expression is reversed by atorvastatin or diacerein treatment (Fig. 6). Since this enzyme has a crucial role in hepatic glucose output, we can suggest that atorvastatin and diacerein, by reducing the downstream TLR4 signaling, can contribute to restoring G6Pase tissue levels and in this regard hepatic glucose production, thus preventing hypoglycemia and improving disease tolerance.

## Discussion

Our data demonstrated that administration of atorvastatin or diacerein improved insulin sensitivity and disease tolerance during peritoneal-induced sepsis. Additionally, these drugs attenuated the increase of inflammatory cytokines and enhanced insulin signaling in the liver and muscle.

Previous studies have shown that there is a clear link between immune and insulin signaling in insulin resistance [40–43]. The activation of serine-kinases IKKβ and JNK occur in metabolic disorders and have an essential role in insulin resistance [43, 44]. Enhanced NF-κB activation is also associated with a more unfortunate outcome in sepsis [45–47]. Furthermore, NF-κB activation increases the transcription of IL-1β, IL-6, and TNF-α [48, 49] and hyperexpression of these mediators may contribute to insulin resistance [50] . The inhibition of NF-κB activation in drug-treated animals provides an explanation for the reduced serum levels of TNF-α and IL-6 and the improvement in the sepsis-induced insulin resistance process.

In both in vitro and in vivo studies, ER stress can activate inflammatory signaling and JNK, resulting in the induction of insulin resistance [11, 51, 52]. In addition to JNK, ER stress also activates IKKβ and this may represent a standard mechanism for the activation of these two counteracting insulin signaling pathways [5]. Our data show that atorvastatin and diacerein reduce ER stress and inhibits JNK phosphorylation and NFκB activation in the liver and muscle of septic rats, indicating that different pathways mediate the beneficial effects of these drugs in improving survival and reducing insulin resistance. Due to the modulation of these inflammatory pathways, these drugs also reduced IRS-1 serine phosphorylation, a tissue marker of insulin resistance. Our data suggest that atorvastatin and diacerein, by attenuating inflammation and sepsis-induced insulin resistance, may be a potential therapy for sepsis through the improvement in insulin sensitivity and signaling in peripheral tissues. On the other hand, a multicenter randomized trial showed that atorvastatin did not result in any differences in length of stay, Sequential Organ Failure Assessment (SOFA) scores or mortalitity in patients with sepsis, when compared to placebo [53]. It is important to emphasize that in this atorvastatin trial, the drug was administered at a moderate dose of 20 mg/day and only given to patients with severe sepsis, indicating that this drug was initiated during a very advanced phase of sepsis. However, this trial did show that prior statin use followed by continuation with atorvastatin during sepsis was associated with improved survival [53]. Taking together these data with our data makes it tempting to speculate that statins may have a beneficial effect in preventing sepsis, and should be continued during the treatment of this condition. Since, in our animal models for the study of sepsis we initiated the statin treatment 3 h after sepsis induction, it is possible that in order to improve survival the atorvastatin should be administered very early and at higher doses, especially in patients not currently taking any type of statin.

The insulin resistance in the liver and peripheral tissues in sepsis is an adaptive response accompanied by mild hyperglycemia, in the early phase. Our data showed that the increase in downstream TLR4 signaling and the induction of ER stress could contribute to the reduced Akt activation and insulin signaling observed in the liver and muscle. Insulin induced IRS/PI3k/Akt activation in muscle has an important role in inducing glucose transporter 4 (GLUT4) translocation and glucose transport in this tissue, and a downregulation of this pathway may explain why insulin resistance is observed in the muscle of septic animals [54]. Along the same lines, the activation of this pathway in the liver reduces neoglucogenesis and hepatic glucose output, and in sepsis, the downregulation of IRS/PI3k/Akt may provide an explanation for the observed increase in hepatic glucose production and hyperglycemia [54].

However, during the evolution of sepsis, the development of hypoglycemia should be prevented, in order to improve disease tolerance [39]. The reduction in insulin signaling in the muscle decreases glucose utilization, towards the goal of preventing hypoglycemia, however this is a limited mechanism. On the other hand, the downregulation in insulin signaling in the liver may not be sufficient enough in preventing hypoglycemia. This is because the downstream TLR4 signaling in the liver, despite its ability to impair insulin signaling, is also able to reduce G6Pase levels, and in this regard decrease the hepatic glucose output. Although we did not measure G6Pase activity in the present study, previous data using heterozygous or homozygous mice with targeted deletion of liver G6Pase showed a close correlation between tissue protein levels and activity [55]. In these mice, this enzymatic deficiency mimics glycogen storage disease type 1a, which is associated with fasting hypoglycemia. This suggests that the reduction in G6Pase protein expression in the liver of septic animals was probably accompained by a reduction in G6Pase activity. In addition, another mechanism recently described [56] that may contribute to induce hypoglycemia in sepsis is an impairment in insulin clearence, also mediated at least in part, by LPS-TLR4. In this regard, these two mechanisms, a decrease in G6Pase and impairment in insulin clearence, probably were offuscated in the past by the simultaneous and predominant effect of insulin resistance. These two driven forces that can contribute to hypglycemia should be prevented, and in this regard our data showed that atorvastatin and diacerein, by reducing lipopolysaccharides (LPS) downstream signaling in the liver, can probably contribute to restoring G6Pase tissue levels and likely its activity, which will ultimately restore hepatic glucose production, and prevent hypoglycemia.

A recent report showed that fasting metabolism is protective against sepsis [57]. However, it is important to maintain at least minimal glucose levels through gluconeogenesis. It is plausible that this fine balance in circulating glucose levels involves opposing mechanisms regulated by TLR4, such as: reduced food intake and peripheral insulin resistance, and endogenous hepatic glucose production. In addition to restoring G6Pase levels and hepatic glucose output, and thus preventing hypoglycemia, these drugs may also prevent hyperglycemia in septic animals by improving glucose utilization in the peripheral tissues, contributing to improved disease tolerance.

Previous data showed that blocking TLR4 signaling with an MD2-TLR4 antagonist had no clear beneficial effects in sepsis, when compared to placebo [15]. As previously mentioned, it is not easy to reconcile a clinical study with patients with a preclinical result in animals, but it is important to re-emphasize that time of treatment and the specific or nonspecific blockage of a single inflammatory pathway are key points in the interpretation of the results. Since there are many inflammatory pathways activated in sepsis, the strategy of merely blocking a single component is likely to be insufficient in controlling the process [58, 59]. Indeed, therapies modulating multiple mediators seem to be more efficacious [2, 58]. The reduced inflammatory response induced by atorvastatin and diacerein improved insulin signaling, and may have also contributed to the increase in survival. The downregulated PI3K/Akt pathways during sepsis may have a role in multiorgan failure, and re-activation of this pathway may ameliorete cardiac dysfunction and brain injury and increase survival in patients with sepsis [53, 60] . Atorvastatin and Diacerein may play a critical role in the protection against sepsis through an improvement in the insulin-induced PI3K/Akt pathway.

## Conclusion

In conclusion, administration of atorvastatin and diacerein to septic animals attenuated ER stress, NF-κB and JNK activation, decreased serum levels of cytokines, increased survival and improved insulin resistance in the liver and muscle. Our results indicate that atorvastatin and diacerein treatments modulate inflammatory pathways and improve disease tolerance through a fine balance in glucose metabolism that might involve G6Pase. Since these two drugs have safety profiles and minimal side effects, we suggest that these drugs may be alternative therapies for the prevention or therapies for the treatment of insulin resistance in sepsis, which could potentially reduce mortality in patients with sepsis.

## Additional file

Additional file 1: **Table S1.** Sequence of the primers used in qPCR analysis. (DOCX 15 kb)

## Abbreviations

NFκB: Factor nuclear kappa B
AKT: Protein kinase B
CLP: Cecal ligation and puncture
ER: Endoplasmic reticulum
G6Pase: glucose-6-phosphatase
GLUT4: glucose transporter 4
IKKβ: inhibitor of nuclear factor kappa-B kinase subunit beta
IL-1β: Interleukin 1 beta
IL6: Interleukin 6
IRE1: Inositol-requiring enzyme 1
IRS-1: Insulin receptor substrate 1
JNK: c-Jun N-terminal kinases
Kitt: Constant rate of glucose disappearance
LPS: Lipopolysaccharide
PERK: Like endoplasmic reticulum kinase
PI3k: Phosphatidylinositide 3-kinases
SOFA: Sequential Organ Failure Assessment
TLR2: Toll-like receptor 2
TLR4: Toll-like receptor 4
TNF-α: Tumor necrosis factor alpha
UPR: Unfolded protein response

## References

[1] Singer M, Deutschman CS, Seymour CW, Shankar-Hari M, Annane D, Bauer M, Bellomo R, Bernard GR, Chiche JD, Coopersmith CM (2016). The third international consensus definitions for Sepsis and septic shock (Sepsis-3). JAMA.

[2] Terblanche M, Almog Y, Rosenson RS, Smith TS, Hackam DG (2006). Statins: panacea for sepsis?. Lancet Infect Dis.

[3] Almog Y (2003). Statins, inflammation, and sepsis: hypothesis. Chest.

[4] Marik PE, Raghavan M (2004). Stress-hyperglycemia, insulin and immunomodulation in sepsis. Intensive Care Med.

[5] van den Berghe G, Wouters P, Weekers F, Verwaest C, Bruyninckx F, Schetz M, Vlasselaers D, Ferdinande P, Lauwers P, Bouillon R (2001). Intensive insulin therapy in critically ill patients. N Engl J Med.

[6] Qu W, Han C, Li M, Zhang J, Jiang Z. Anti-TNF-α antibody alleviates insulin resistance in rats with sepsis-induced stress hyperglycemia. J Endocrinol Invest. 2018;41(4):455-63. 10.1007/s40618-017-0742-7. Epub 2017 Oct 13. 10.1007/s40618-017-0742-7 29030784

[7] Umbarawan Y, Syamsunarno MRAA, Obinata H, Yamaguchi A, Sunaga H, Matsui H, Hishiki T, Matsuura T, Koitabashi N, Obokata M (2017). Robust suppression of cardiac energy catabolism with marked accumulation of energy substrates during lipopolysaccharide-induced cardiac dysfunction in mice. Metabolism.

[8] Cao J, Peng J, An H, He Q, Boronina T, Guo S, White MF, Cole PA, He L (2017). Endotoxemia-mediated activation of acetyltransferase P300 impairs insulin signaling in obesity. Nat Commun.

[9] Delile E, Nevière R, Thiébaut PA, Maupoint J, Mulder P, Coquerel D, Renet S, Rieusset J, Richard V, Tamion F (2017). Reduced insulin resistance contributes to the beneficial effect of protein tyrosine phosphatase-1B deletion in a mouse model of Sepsis. Shock.

[10] Illuri VD, Layden BT, Aleppo G (2016). Extreme insulin resistance in critically ill patient with Sepsis. Clin Diabetes.

[11] Hotamisligil GS (2010). Endoplasmic reticulum stress and the inflammatory basis of metabolic disease. Cell.

[12] Rorato R, Borges BC, Uchoa ET, Antunes-Rodrigues J, Elias CF, Elias LLK. LPS-induced low-grade inflammation increases hypothalamic JNK expression and causes central insulin resistance irrespective of body weight changes. Int J Mol Sci. 2017;18(7). doi: 10.3390/ijms18071431. 10.3390/ijms18071431 PMC5535922 28677618

[13] Wheeler AP, Bernard GR (1999). Treating patients with severe sepsis. N Engl J Med.

[14] From the bench to the bedside: the future of sepsis research. Executive summary of an American College of Chest Physicians, National Institute of Allergy and Infectious Disease, and National Heart, Lung, and Blood Institute Workshop. Chest. 1997;111:744-53. https://www.ncbi.nlm.nih.gov/pubmed/9118716. 9118716

[15] Opal SM, Laterre PF, Francois B, LaRosa SP, Angus DC, Mira JP, Wittebole X, Dugernier T, Perrotin D, Tidswell M (2013). Effect of eritoran, an antagonist of MD2-TLR4, on mortality in patients with severe sepsis: the ACCESS randomized trial. JAMA.

[16] Spitzer AL, Harris HW (2006). Statins attenuate sepsis. Surgery.

[17] Mortensen EM, Restrepo MI, Copeland LA, Pugh JA, Anzueto A, Cornell JE, Pugh MJ (2007). Impact of previous statin and angiotensin II receptor blocker use on mortality in patients hospitalized with sepsis. Pharmacotherapy.

[18] Liappis AP, Kan VL, Rochester CG, Simon GL (2001). The effect of statins on mortality in patients with bacteremia. Clin Infect Dis.

[19] Tleyjeh IM, Kashour T, Hakim FA, Zimmerman VA, Erwin PJ, Sutton AJ, Ibrahim T (2009). Statins for the prevention and treatment of infections: a systematic review and meta-analysis. Arch Intern Med.

[20] Frost FJ, Petersen H, Tollestrup K, Skipper B (2007). Influenza and COPD mortality protection as pleiotropic, dose-dependent effects of statins. Chest.

[21] Kruger P, Fitzsimmons K, Cook D, Jones M, Nimmo G (2006). Statin therapy is associated with fewer deaths in patients with bacteraemia. Intensive Care Med.

[22] Majumdar SR, McAlister FA, Eurich DT, Padwal RS, Marrie TJ (2006). Statins and outcomes in patients admitted to hospital with community acquired pneumonia: population based prospective cohort study. BMJ.

[23] Mortensen EM, Restrepo MI, Anzueto A, Pugh J (2005). The effect of prior statin use on 30-day mortality for patients hospitalized with community-acquired pneumonia. Respir Res.

[24] Thomsen RW, Hundborg HH, Johnsen SP, Pedersen L, Sørensen HT, Schønheyder HC, Lervang HH (2006). Statin use and mortality within 180 days after bacteremia: a population-based cohort study. Crit Care Med.

[25] Smith GN, Myers SL, Brandt KD, Mickler EA, Albrecht ME (1999). Diacerhein treatment reduces the severity of osteoarthritis in the canine cruciate-deficiency model of osteoarthritis. Arthritis Rheum.

[26] Moore AR, Greenslade KJ, Alam CA, Willoughby DA (1998). Effects of diacerhein on granuloma induced cartilage breakdown in the mouse. Osteoarthr Cartil.

[27] Nicolas P, Tod M, Padoin C, Petitjean O (1998). Clinical pharmacokinetics of diacerein. Clin Pharmacokinet.

[28] Pelletier JP, Lajeunesse D, Reboul P, Mineau F, Fernandes JC, Sabouret P, Martel-Pelletier J (2001). Diacerein reduces the excess synthesis of bone remodeling factors by human osteoblast cells from osteoarthritic subchondral bone. J Rheumatol.

[29] Pelletier JP, Jovanovic D, Fernandes JC, Manning P, Connor JR, Currie MG, Di Battista JA, Martel-Pelletier J (1998). Reduced progression of experimental osteoarthritis in vivo by selective inhibition of inducible nitric oxide synthase. Arthritis Rheum.

[30] Del Rosso M, Fibbi G, Magnelli L, Pucci M, Dini G, Grappone C, Caldini R, Serni U, Colombo F, Borella F (1990). Modulation of urokinase receptors on human synovial cells and osteoarthritic chondrocytes by diacetylrhein. Int J Tissue React.

[31] Mendes AF, Caramona MM, de Carvalho AP, Lopes MC (2002). Diacerhein and rhein prevent interleukin-1beta-induced nuclear factor-kappaB activation by inhibiting the degradation of inhibitor kappaB-alpha. Pharmacol Toxicol.

[32] Rittirsch D, Huber-Lang MS, Flierl MA, Ward PA (2009). Immunodesign of experimental sepsis by cecal ligation and puncture. Nat Protoc.

[33] Clarke RM, O'Connell F, Lyons A, Lynch MA (2007). The HMG-CoA reductase inhibitor, atorvastatin, attenuates the effects of acute administration of amyloid-beta1-42 in the rat hippocampus in vivo. Neuropharmacology.

[34] Dostal LA, Whitfield LR, Anderson JA (1996). Fertility and general reproduction studies in rats with the HMG-CoA reductase inhibitor, atorvastatin. Fundam Appl Toxicol.

[35] Tamura T, Shirai T, Kosaka N, Ohmori K, Takafumi N (2002). Pharmacological studies of diacerein in animal models of inflammation, arthritis and bone resorption. Eur J Pharmacol.

[36] Carvalho-Filho MA, Ueno M, Hirabara SM, Seabra AB, Carvalheira JB, de Oliveira MG, Velloso LA, Curi R, Saad MJ (2005). S-nitrosation of the insulin receptor, insulin receptor substrate 1, and protein kinase B/Akt: a novel mechanism of insulin resistance. Diabetes.

[37] Bonora E, Targher G, Alberiche M, Bonadonna RC, Saggiani F, Zenere MB, Monauni T, Muggeo M (2000). Homeostasis model assessment closely mirrors the glucose clamp technique in the assessment of insulin sensitivity: studies in subjects with various degrees of glucose tolerance and insulin sensitivity. Diabetes Care.

[38] Thirone AC, Carvalheira JB, Hirata AE, Velloso LA, Saad MJ (2004). Regulation of Cbl-associated protein/Cbl pathway in muscle and adipose tissues of two animal models of insulin resistance. Endocrinology.

[39] Weis S, Carlos AR, Moita MR, Singh S, Blankenhaus B, Cardoso S, Larsen R, Rebelo S, Schäuble S, Del Barrio L (2017). Metabolic adaptation establishes disease tolerance to Sepsis. Cell.

[40] Shoelson SE, Lee J, Goldfine AB (2006). Inflammation and insulin resistance. J Clin Invest.

[41] Hotamisligil GS (2006). Inflammation and metabolic disorders. Nature.

[42] Dandona P, Aljada A, Bandyopadhyay A (2004). Inflammation: the link between insulin resistance, obesity and diabetes. Trends Immunol.

[43] Arkan MC, Hevener AL, Greten FR, Maeda S, Li ZW, Long JM, Wynshaw-Boris A, Poli G, Olefsky J, Karin M (2005). IKK-beta links inflammation to obesity-induced insulin resistance. Nat Med.

[44] Hirosumi J, Tuncman G, Chang L, Gorgun CZ, Uysal KT, Maeda K, Karin M, Hotamisligil GS (2002). A central role for JNK in obesity and insulin resistance. Nature.

[45] Arcaroli J, Silva E, Maloney JP, He Q, Svetkauskaite D, Murphy JR, Abraham E (2006). Variant IRAK-1 haplotype is associated with increased nuclear factor-kappaB activation and worse outcomes in sepsis. Am J Respir Crit Care Med.

[46] Arnalich F, Garcia-Palomero E, López J, Jiménez M, Madero R, Renart J, Vázquez JJ, Montiel C (2000). Predictive value of nuclear factor kappaB activity and plasma cytokine levels in patients with sepsis. Infect Immun.

[47] Yang KY, Arcaroli JJ, Abraham E (2003). Early alterations in neutrophil activation are associated with outcome in acute lung injury. Am J Respir Crit Care Med.

[48] Baldwin AS (1996). The NF-kappa B and I kappa B proteins: new discoveries and insights. Annu Rev Immunol.

[49] Baeuerle PA, Baltimore D, NF-kappa B (1996). Ten years after. Cell.

[50] Peraldi P, Hotamisligil GS, Buurman WA, White MF, Spiegelman BM (1996). Tumor necrosis factor (TNF)-alpha inhibits insulin signaling through stimulation of the p55 TNF receptor and activation of sphingomyelinase. J Biol Chem.

[51] Zhang X, Zhang G, Zhang H, Karin M, Bai H, Cai D (2008). Hypothalamic IKKbeta/NF-kappaB and ER stress link overnutrition to energy imbalance and obesity. Cell.

[52] Deng J, Lu PD, Zhang Y, Scheuner D, Kaufman RJ, Sonenberg N, Harding HP, Ron D (2004). Translational repression mediates activation of nuclear factor kappa B by phosphorylated translation initiation factor 2. Mol Cell Biol.

[53] Tang G, Yang H, Chen J, Shi M, Ge L, Ge X, Zhu G (2017). Metformin ameliorates sepsis-induced brain injury by inhibiting apoptosis, oxidative stress and neuroinflammation via the PI3K/Akt signaling pathway. Oncotarget.

[54] Boucher J, Kleinridders A, Kahn CR. Insulin receptor signaling in normal and insulin-resistant states. Cold Spring Harb Perspect Biol. 2014;6(1). doi: 10.1101/cshperspect.a009191. 10.1101/cshperspect.a009191 PMC3941218 24384568

[55] Mutel E, Abdul-Wahed A, Ramamonjisoa N, Stefanutti A, Houberdon I, Cavassila S, Pilleul F, Beuf O, Gautier-Stein A, Penhoat A (2011). Targeted deletion of liver glucose-6 phosphatase mimics glycogen storage disease type 1a including development of multiple adenomas. J Hepatol.

[56] Hagar JA, Edin ML, Lih FB, Thurlow LR, Koller BH, Cairns BA, Zeldin DC, Miao EA (2017). Lipopolysaccharide potentiates insulin-driven hypoglycemic shock. J Immunol.

[57] Wang A, Huen SC, Luan HH, Yu S, Zhang C, Gallezot JD, Booth CJ, Medzhitov R (2016). Opposing effects of fasting metabolism on tissue tolerance in bacterial and viral inflammation. Cell.

[58] Marshall JC (2004). Sepsis: current status, future prospects. Curr Opin Crit Care.

[59] Böhrer H, Qiu F, Zimmermann T, Zhang Y, Jllmer T, Männel D, Böttiger BW, Stern DM, Waldherr R, Saeger HD (1997). Role of NFkappaB in the mortality of sepsis. J Clin Invest.

[60] Li C, Hua F, Ha T, Singh K, Lu C, Kalbfleisch J, Breuel KF, Ford T, Kao RL, Gao M (2012). Activation of myocardial phosphoinositide-3-kinase p110α ameliorates cardiac dysfunction and improves survival in polymicrobial sepsis. PLoS One.

