# Supplementary material for: Atorvastatin and diacerein reduce insulin resistance and increase disease tolerance in rats with sepsis
Source: J Inflamm (Lond). 2018 May 9;15:8. doi: 10.1186/s12950-018-0184-9 (PMC5944072; doi:10.1186/s12950-018-0184-9)
Supplement: Supplementary file 1 — Table S1. Sequence of the primers used in qPCR analysis. (DOCX 15 kb) [file 12950_2018_184_MOESM1_ESM.docx]

Table 1 Sequence of the primers used in qPCR analysis

| **Gene** | Sequence 5’-3’ | |
| --- | --- | --- |
|  | Forward | Reverse |
| **IRE1 (IRN1)** | AAGGCAAGAGCAAGCTGAACTAC | CGTGATTCCTGCTGATTCGG |
| **PERK (EIF2AK3)** | ATTATTCCTCTGCCGACGATCA | TTGAAAGCGCACCATTACTGTAT |
| **Actb**  **(β-actin)** | GCAATGAGCGGTTCCGATG | TAGTTTCATGGATGCCACAGGAT |
